# Neural Responses to Truth Telling and Risk Propensity under Asymmetric Information

**DOI:** 10.1371/journal.pone.0137014

**Published:** 2015-09-01

**Authors:** Hideo Suzuki, Masaya Misaki, Frank Krueger, Jerzy Bodurka

**Affiliations:** 1 Laureate Institute for Brain Research, Tulsa, OK, United States of America; 2 Molecular Neuroscience Department, George Mason University, Fairfax, VA, United States of America; 3 College of Engineering, University of Oklahoma, Norman, OK, United States of America; University Of Cambridge, UNITED KINGDOM

## Abstract

Trust is multi-dimensional because it can be characterized by subjective trust, trust antecedent, and behavioral trust. Previous research has investigated functional brain responses to subjective trust (e.g., a judgment of trustworthiness) or behavioral trust (e.g., decisions to trust) in perfect information, where all relevant information is available to all participants. In contrast, we conducted a novel examination of the patterns of functional brain activity to a trust antecedent, specifically truth telling, in asymmetric information, where one individual has more information than others, with the effect of varying risk propensity. We used functional magnetic resonance imaging (fMRI) and recruited 13 adults, who played the Communication Game, where they served as the “Sender” and chose either truth telling (true advice) or lie telling (false advice) regarding the best payment allocation for their partner. Our behavioral results revealed that subjects with recreational high risk tended to choose true advice. Moreover, fMRI results yielded that the choices of true advice were associated with increased cortical activation in the anterior rostral medial and frontopolar prefrontal cortices, middle frontal cortex, temporoparietal junction, and precuneus. Furthermore, when we specifically evaluated a role of the bilateral amygdala as the region of interest (ROI), decreased amygdala response was associated with high risk propensity, regardless of truth telling or lying. In conclusion, our results have implications for how differential functions of the cortical areas may contribute to the neural processing of truth telling.

## Introduction

Trust is characterized by three constructs: subjective trust, behavioral trust, and trust antecedents [1]. *Subjective trust* is an internal state of cognitive and social processing of trust (e.g., a perception/evaluation of others as trustworthy or not), which results from a *trust antecedent* (a psychological precursor leading to trust) and in *behavioral trust* (an overt action reflecting trust). The interplay of these constructs directly or indirectly optimizes group performance [2–7] and helps accomplish social capital in practical settings, such as the treatment success in psychotherapy [8, 9], therefore it is important to study subjective trust, behavioral trust, and trust antecedents.

Subjective trust may be determined by social and cognitive processing in specific brain areas. For example, the amygdala is involved in trustworthiness judgments of neutral faces [10–15]. The anterior insular cortex and dorsal anterior cingulate cortex, contributing to the salience network [16, 17], may also serve our ability to perceive salient, affective tones in communication and to preconceive trustworthiness. Furthermore, the dorsolateral prefrontal cortex and posterior cingulate cortex, involved in the central-executive network [16], may alternatively serve to scrutinize trustworthiness.

Behavioral trust (e.g., decisions to trust) has also been examined in the context of an economic exchange paradigm. In investment games, [18], subjects show increased functional activation in the caudate head during their repayments to a benevolent investor relative to a malevolent investor [19] and monetary gain following their investment [20]. Moreover, damage to the ventromedial prefrontal cortex [21] and amygdala [22, 23] is associated with a benevolent type of investor strategy (i.e., investing more) for their partner, suggesting the important functions of the ventromedial prefrontal cortex and amygdala in social vigilance and prospection in decisions to trust. Furthermore, in voluntary trust games, the decisions to trust as the first move is related to increased activation in the paracingulate cortex/anterior rostral medial prefrontal cortex (armPFC), and septal area [24]. With the same data, further analysis shows that the armPFC and anterior insular cortex are involved in shared neurocircuitry of decisions to trust and reciprocate, while the frontopolar cortex (fpPFC) and temporoparietal junction (TPJ) are exclusively associated with decisions to initiate trusting [25]. These findings indicate that behavioral trust may be associated with functions of the caudate, amygdala, and some cortical areas.

While previous literature had addressed the patterns of brain activity concerning subjective trust and behavioral trust, to our knowledge, few studies have focused on neural antecedents of trust, especially under asymmetric information. *Asymmetric information* refers to an economic/social communication where one participant has better or superior information than the others [26–28], as opposed to *perfect information*, where relevant information is equally available to all participants. The most important trust antecedent under asymmetric information is truth telling behavior initiated by those who have better information [26]. For example, in a psychotherapeutic interaction, a mental health practitioner, who is knowledgeable of the diagnosis and treatment of mental health, communicates with a patient, who needs professional advice and help. If a patient realizes that the mental health practitioner tells the truth and is convinced of the appropriateness of the practitioner’s treatment, the patient would be willing to follow the practitioner’s advice and stay in the treatment for a sufficiently long time, which builds therapeutic alliance and promotes the efficiency of interpersonal therapeutic treatment [8, 9, 29–31] and medication treatment [32, 33]. In this way, well informed participant’s truth telling under asymmetric information is crucial to precede trust (i.e., trust antecedent), and the current study aimed to examine neural biomarkers for truth telling. Notably, while previous studies have mentioned functional brain activity to decisions to trust [24, 25], which may be similar to brain activity in a less informed participant who needs to decide either to trust or not under asymmetric information, few studies have addressed brain functions in a well-informed participant’s truth telling over lying. Although one relevant study has shown that cognitive functions of the anterior cingulate cortex, dorsolateral prefrontal cortex, and ventromedial prefrontal cortex are involved in lying behavior under impersonal context [34], functional brain activity during truth telling under asymmetric information has not been well investigated.

In addition to truth telling, risk propensity might also serve as a trust antecedent in asymmetric information, because risk propensity is essential to determine whether a well informed participant overcomes potential risks for being exploited [35] and betrayed [36] following truth telling. Therefore, the present study also focused on functional brain activity in relation to risk propensity.

Therefore, the aims of our study were to examine neuro-biomarkers for the interaction between truth telling and risk propensity under asymmetric information, which would be important to advance our understanding of how interpersonal trust is formed. To manipulate asymmetric information, we used the Communication Game, designed as a paradigm to assess truth-telling behavior in asymmetric information [26, 37, 38]. In the Communication Game, one player, called the *Sender*, is informed of three payoff options—one leading to a high payoff for oneself but a medium payoff for the partner, another one to a medium payoff for oneself but a high payoff for the partner, and the other one to low payoffs for both—and decides to tell the partner either the truth (“true advice”) or lie (“false advice”) regarding the high payoff for the partner. For analyzing neuro-biomarkers, we used functional magnetic resonance imaging (fMRI) and performed whole-brain analysis. In addition to the whole-brain analysis, we specifically focused on amygdala activity because the amygdala might commonly active in both trust-related behavior and risk-taking behavior. That is, amygdala functions contribute to social processing of evaluating trustworthiness of faces in healthy subjects [10–15], and amygdala activity to ratings of trustworthiness was reduced in patients with schizophrenia and autistic spectrum disorder [39, 40], who often show difficulty in social judgment, including trustworthiness [41–43]. Although the social evaluation of trustworthiness is not equivalent to truth telling, it would be intriguing to examine whether amygdala functions extend to the psychological processing of truth telling. On the other hand, the amygdala is also associated with vigilance [12, 44, 45] and risk-taking behavior [46, 47]. For instance, patients with substance abuse [48, 49] show reduced amygdala activity to risky decision-making as compared to healthy controls. This suggests that their amygdala may be insensitive to risk decision making, encouraging the psychiatric patients to show risk-taking behavior. Because of possible relations of truth telling and risk propensity with amygdala functions, the present study focused on the amygdala as the region-of-interest (ROI) analysis.

We hypothesized that (1) the Sender with high risk propensity would tell the truth more often than one with low risk propensity, because high risk-takers tend to behave in trusting manners [1, 50] and (2) the Sender would show functional brain changes, especially the amygdala, during the choices of true advice (relative to false advice) in asymmetric information, while this relationship depended on the degree of risk propensity.

## Methods

### Participants

The study was conducted at the Laureate Institute for Brain Research. The research protocol was approved by the Western Institutional Review Board. Human research in this study was conducted according to the principles expressed in the Declaration of Helsinki. All fourteen healthy adults were recruited from Tulsa metro area. Study subjects gave written informed consent to participate and received financial compensation. None of them showed any clinically significant physical illness, a history of traumatic brain injury, severe vision/hearing loss, or any Axis I psychiatric disorder based on the Structured Clinical Interview for DSM-IV-TR Axis I Disorders (SCID-I/NP) [51]. One of them was, however, excluded because we faced a technical problem when this subject was scanned. As a result, a total of *N* = 13 subjects (8 female), whose age ranged from 21 to 31, were included in the data analyses. Table 1 shows some background characteristics of our sample. After the study, the subjects received financial compensation for their study participation.

**Table 1:** Background Characteristics of the Sample (*N* = 13).

Variable	Descriptive statistics
**Mean age in years *(SD*)**	25 (4)
**Sex** ^1^	
**Female**	8
**Male**	5
**Educational level** ^1^	
**Some college/technical school (at least one year)**	6
**College graduate**	6
**Graduate professional training (master or above)**	1
**Mean RTI (*SD*)**	20.46 (5.46)
**Mean percentage of advice sent as the Sender**	
**True advice**	67.8% (22.7%)
**False advice**	31.6% (22.7%)

^1^Data are presented as frequency.

### Risk propensity

Risk Taking Inventory (RTI) was used to assess the frequency of risk-taking behavior in everyday life, based on a five-point scale (1 = never; 5 = very often) [52]. The RTI is a self-report questionnaire, where six dimensions of each of current and past risk-taking behaviors during adulthood were measured. These dimensions included recreational risks (e.g., rock-climbing, scuba diving), health risks (e.g., smoking, poor diet, high alcohol consumption), career risks (e.g., quitting a job without another to go to), financial risks (e.g., gambling, risky investments), safety risks (e.g., fast driving, city cycling without a helmet), and social risks (e.g., standing for election, publicly challenging a rule or decision). The total score and each subscore were 60 and 10 at maximum, respectively, and a higher score indicated higher risk propensity.

### Communication Game

**Fig 1:**
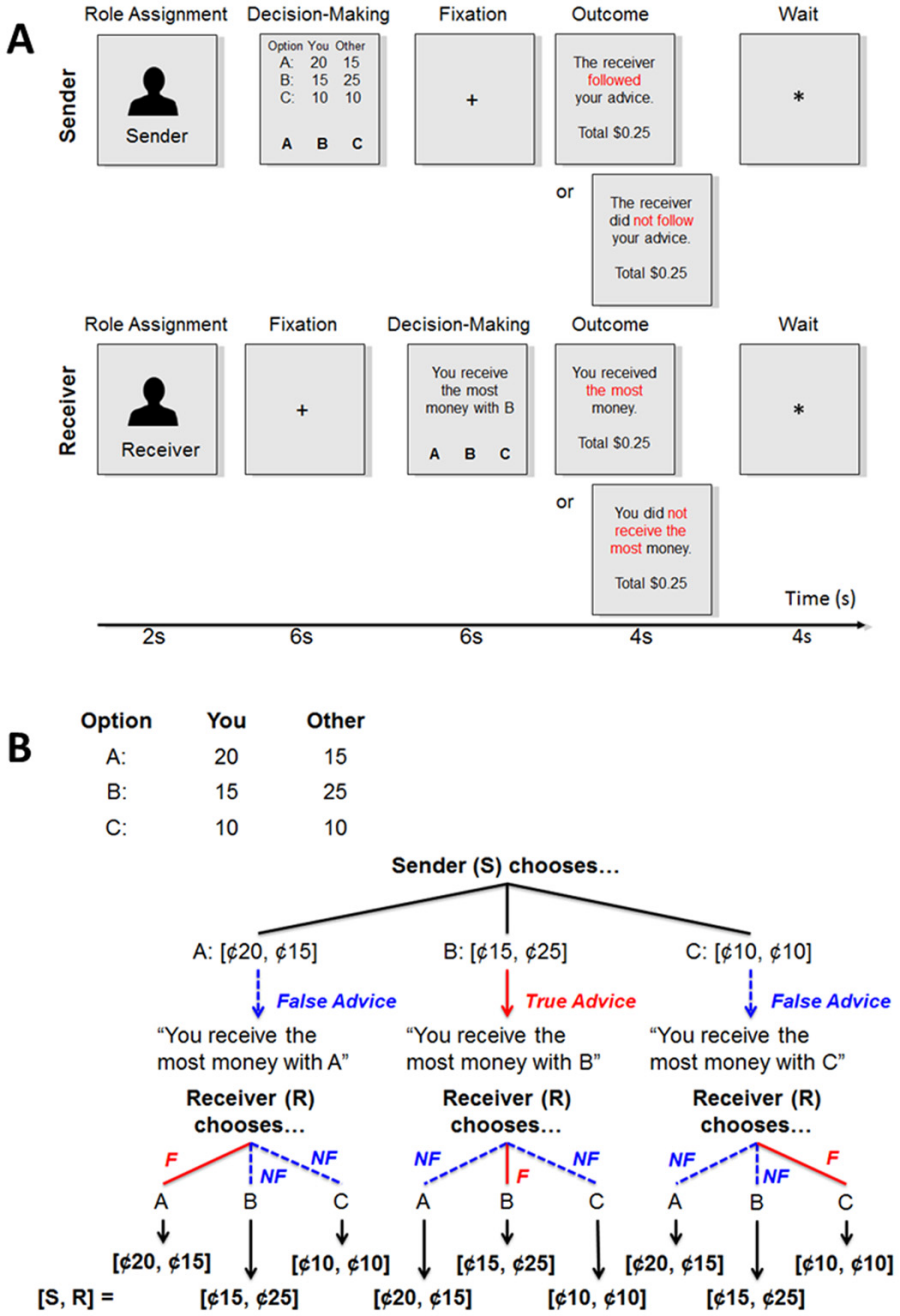
Experimental design. (A) Timeline for a single Communication Game. A subject was assigned to either the Sender or Receiver. The Sender viewed three payment pairs and was instructed to choose true advice (the pair allocating the most money to the Receiver) or false advice (the pairs allocating less money to the Receiver) for the Receiver. In contrast, the Receiver could not view the payment pairs. Instead, the Receiver was instructed to choose one pair based on the Sender’s advice. The Receiver’s choice determined the final allocation of money to each player. Finally, both the Sender and Receiver could view information about whether their partner was trustworthy or trusting. (B) An example of three payment pairs which the Sender might view during a Communication Game trial. In this example, the Sender (S) delivered true advice if she/he chose option B; the Sender delivered false advice if she/he chose options A or C. Then, the Sender’s choice of advice was presented to the Receiver (R), and the Receiver did follow (“F”) or did not follow (“NF”) the advice. The Receiver’s choice determined how much S and R gained.

As the fMRI task, the Communication Game was used to manipulate sequential situations where individuals faced conflicts between their own financial gain and their partner’s gain in asymmetric information [26, 37, 38]. The Communication Game was programmed using the Willow experimental economics software framework (George Mason University, Fairfax, VA). In this game, a pair of two players interacted with each other to determine payoffs to each player. In the present study, although subjects were informed that they would interact with either human or computer-programming player, the player partner was actually performed by computer programming across all trials; the player partner was programmed to choose true/false advice or to follow/disregard advice with varying probabilities, depending on subjects’ response at the previous trial (for details about the probabilities, see S1 Table). After the MRI study, all subjects were debriefed and informed that they indeed interacted with a computer-programming player. Fig 1A illustrates the flow of each Communication Game trial. Each subject played the role of either the *Sender* or *Receiver*; when the subject was assigned to one of the roles, the partner (programmed by computer) was automatically assigned to the other role. These roles were switched between two players across trials.

When subjects were designated to the Sender, they were presented with three payoff pairs each of which indicated how much was allocated to each player (see Fig 1B as an example). These pairs included (A) $0.20 given to oneself and $0.15 given to the partner, (B) $0.15 given to oneself and $0.25 given to the partner, and (C) $0.10 to given to oneself and the partner, although these pairs were shuffled every trial (e.g., the pair of $0.20 and $0.15 belongs to option A sometimes and option B or C other times). The Sender was instructed to choose one best pair allocating the most money to the partner (i.e., Receiver) within 6 sec (e.g., option B in Fig 1B). However, the Sender was also instructed that she/he could try to deceive the partner to gain more money for her-/himself (e.g., option A in Fig 1B) or to make even allocation (e.g., option C in Fig 1B). That is, in such a conflict situation, the Sender could deliver true advice (i.e., telling a truth) or false advice (i.e., telling a lie) to the partner. Note that the pair of $0.10 and $0.10 was included because it rules out the possibility that the Sender tactically chose true advice to spike the partner’s expected choice of not following the advice and to gain her/his own benefit, rather than choosing true advice as being honest for the partner [26].

In contrast, when subjects were designated as the Receiver, they waited for 6 sec during which the partner (i.e., Sender) chose true or false advice. Then the Receiver was presented with a message showing which payoff was advised by the partner. For example, if the partner selected option B, the Receiver viewed a message, “You receive the most money with B.” Note that the information about the payoff pairs was not disclosed to the Receiver. In this way, the Sender had more pieces of information (i.e., available payoff pairs) than the Receiver, manipulating information asymmetry between two players. The Receiver had to choose one option based on only the partner’s advice within 6 sec. If the Receiver trusted the partner, the Receiver was expected to choose the option according to the advice; otherwise, the Receiver would choose the option against the advice.

Importantly, the Receiver’s choice determined the final payoff. Then, both the Sender and Receiver reviewed their partner’s choice behavior. That is, the Sender was informed whether or not the Receiver followed her/his advice. On the other hand, the Receiver was informed whether she/he gained the most money. When the next trial started, a subject’s role (either Sender or Receiver) was determined randomly, and the payoff pairs were shuffled across options A, B, and C.

Although we collected data of subjects’ responses as both the Sender and Receiver, the present study focused on only subjects’ responses as the Sender. This was because the present study specifically aimed to examine functional brain responses to the choices of true advice/truth telling as a trust antecedent in asymmetric information.

### Procedure and MRI data acquisition

Prior to scans, subjects were asked to complete the RTI and then given instruction of how to play the Communication Game with their partner. They were also told that they could get money based on their task performances, although all subjects ultimately received $20.00 regardless of their actual performances. Some subjects met a confederate face-to-face to make them believe that a potential player partner existed, but other subjects did not meet anyone. Although our analyses mixed these subjects, a previous study revealed that functional brain responses during economic exchange games were not affected by a prior personal encounter with a player partner [53].

Neuroimaging data were acquired on Discovery MR750 3 Tesla MRI whole-body MRI scanner (General Electric Healthcare Technologies, Waukesha, WI) equipped with 32-channel MRI receiver capable of conducting, in real time, fMRI with parallel imaging such as Sensitivity Encodings (SENSE) [54]. An 8-element receive-only head coil array was used for fMRI signal reception. During each fMRI scan, physiological cardiac and respiratory waveforms were simultaneously acquired (40 Hz sampling rate). The cardiac waveform was measured using a photoplethysmograph pad with an infra-red emitter, pulse oximetry from the subject’s left index finger; respiration waveform was measured using a pneumatic respiration belt. MRI session involved a localizer scan for prescribing the following anatomical and functional scans, a 5-minute anatomical scan for localizing and aligning functional scans, a 7.5-minute resting-state functional scan, and four 9-minute Communication-Game-related functional scans. For the anatomical scan, one three-dimensional T1-weighted magnetization prepared rapid gradient echo (MPRAGE) scan with SENSE (TR/TE = 5/1.92 ms, inversion/delay time TI/TD = 725/1400 ms, flip angle = 8°, FOV = 240 mm, axial slices per slab = 128, image matrix = 256 × 256, voxel volume = 0.94 × 0.94 × 1.20 mm^3^, acceleration factor R = 2, sampling band-width = 31.3 kHz) was acquired in the axial plane. For the functional scans, blood-oxygen-level-dependent (BOLD) images were acquired with a T2*-weighted single-shot gradient-recalled echo-planer imaging (EPI) sequence (TR/TR = 2000/25 ms, flip angle = 78°, FOV = 240 mm, acquisition matrix = 96 × 96 reconstructed to image matrix of 128 × 128, voxel volume = 1.875 × 1.875 × 2.900 mm^3^, axial slices per volume = 34, number of volumes = 263, SENSE acceleration factor R = 2 in the phase encoding (anterior-posterior commissure plane) direction, sampling bandwidth = 250kHz).

Each of Communication Game functional scans consisted of 24 trials; subjects were assigned to the Sender in 12 trials and the Receiver in the other 12 trials. Since our focus was to analyze functional brain responses to the choices of true advice, our data analyses used only imaging data acquired during Communication Game trials where subjects acted as the Sender.

The total amount of time for MRI scans was less than 2 hours. During all functional scans, simultaneous electroencephalography (EEG) recordings were additionally performed using a 32-channel MR-compatible EEG system (Brain Products GmbH), although EEG data were not used in the present study.

### fMRI data preprocessing

Analysis of Functional Neuroimages (AFNI) [55] was used for fMRI data analyses. The first four volumes in each scan were excluded from the data analysis to avoid T1 equilibrium effect. Physiological noise correction was conducted to suppress cardiorespiratory signal modulations by utilizing the cardiac and respiratory waveforms recorded during scans and employing the RETROICOR [56] implementation in AFNI. Further, slice timing correction and volume registration to the first volume were applied. The EPI images were spatially transformed to the Talairach and Tornoux [57] template brain using Advanced Normalization Tools (ANTS, http://picsl.upenn.edu/software/ants/) with the Symmetric Normalization (SyN) method [58]. SyN is an algorithm for applying a bi-directional diffeomorphism, with maximizing a cross-correlation metric. The normalized image was resampled to 1.875 mm^3^ isotropic voxel. Spatial smoothing was applied by convolving a 4.0mm-based full width at half maximum (FWHM) Gaussian kernel. The signal time course was scaled to percent change relative to the mean signal across time in each voxel. General linear model (GLM) analysis was conducted to evaluate functional brain activation. The design matrix included modeled responses for showing a role assignment image, the Sender’s decision-making, wait as the Sender, outcome message for the Sender, the Receiver’s decision-making, wait as the Receiver, and outcome message for the Receiver (see Fig 1A). The response models were constructed by convolving boxcar functions for each event time course with a Gamma function model of hemodynamic response. In order to incorporate within-subject response variability into our group analysis, we estimated the trial-wise response of the Sender’s decision-making by modeling each trial response independently [59]. This yielded beta series of trial-wise responses of the Sender’s decision-making. In addition to these task-related regressors, six motion parameters (shifts in x, y, and z directions and roll, pitch, yaw rotations), their temporal derivatives, and 4th-order polynomial regressors for modeling slow frequency noises were included in the design matrix.

### Statistical analysis

For the behavioral analysis (S1 Dataset), we first checked whether the number of choosing true advice was related to age (using Spearman correlation), gender, and educational levels (using one-way ANOVA). Then Spearman’s rank correlation was used to test the associations between the total RTI score and the number of instances choosing true advice. In addition, the other Spearman correlations between each of the RTI subscores (i.e., recreational risks, health risks, career risks, financial risks, safety risks, and social risks) and the number of instances choosing true advice was employed, with Bonferroni-typed adjustments for multiple comparisons.

For fMRI analysis (S2 Dataset), we employed linear mixed-effects models (LME) analysis [60] and treated subject variability as a random-effect variable in the R statistical computing language and environment [61]. The beta series of the Sender’s decision-making activation was entered as a dependent variable. The advice choice (the choices of true advice relative to false advice), the standardized RTI score, and their interaction were entered as fixed effects and subject variability as a random effect. The statistical parametric map was thresholded with voxel-wise p < 0.005 and then cluster size ≥ 65 with first-nearest neighbor clustering for family-wise error correction. Cluster size threshold was determined by a Monte-Carlo simulations using 3dClustSim in AFNI with smoothness of 5.31 × 5.43 × 5.14 mm, which was estimated from a residual image of the LME analysis.

In addition, the amygdala ROIs in both hemispheres were created from the Talairach and Tornoux (57) atlas. This ROI analysis (S3 Dataset) was performed based on our *a priori* hypothesis that amygdala function would be commonly associated with both truth-telling and risk propensity, as discussed earlier. The ROIs were resampled to the resolution of the normalized functional image, clipping off voxels that were less than 50% occupied. The beta values within the ROIs were averaged for each of the Sender’s decision-making epochs using 3dROIstats in AFNI. Then three-way repeated measures ANCOVA was used, with the average BOLD signal within each ROI as the dependent variable, the advice choice and hemisphere as the within-subjects variables, and the RTI score as the between-subjects covariate.

## Results

### Behavioral analysis

The number of instances choosing true advice was not affected by age (*ρ*(11) = −0.05, *p* > 0.05), gender (*F*(1,11) = 1.55, *p* > 0.05), or educational level (*F*(2,10) = 2.68, *p* > 0.05). Moreover, Table 2 shows that the number of instances choosing true advice was not significantly correlated with the total RTI score. Nevertheless, in the subscale analyses, interestingly, the number of instances choosing true advice was correlated only with recreational risks; the Senders with high recreational risks were likely to deliver more true advice than those with low recreational risks. The other dimensions of risk-taking behaviors were not correlated with the number of instances choosing true advice.

**Table 2:** Spearman’s Rank Correlations between RTI Scores and the Number of the choices of True Advice (*N* = 13).

	Total	Recreational	Health	Career	Financial	Safety	Social
**Number of choices of true advice**	0.38	0.74*	0.04	−0.08	0.16	0.21	0.53

*Note*: Values indicate Spearman’s correlation coefficients, with *df* = 11.

*p < .05 with the Bonferroni correction.

### Functional MRI analysis of the whole brain

**Fig 2:**
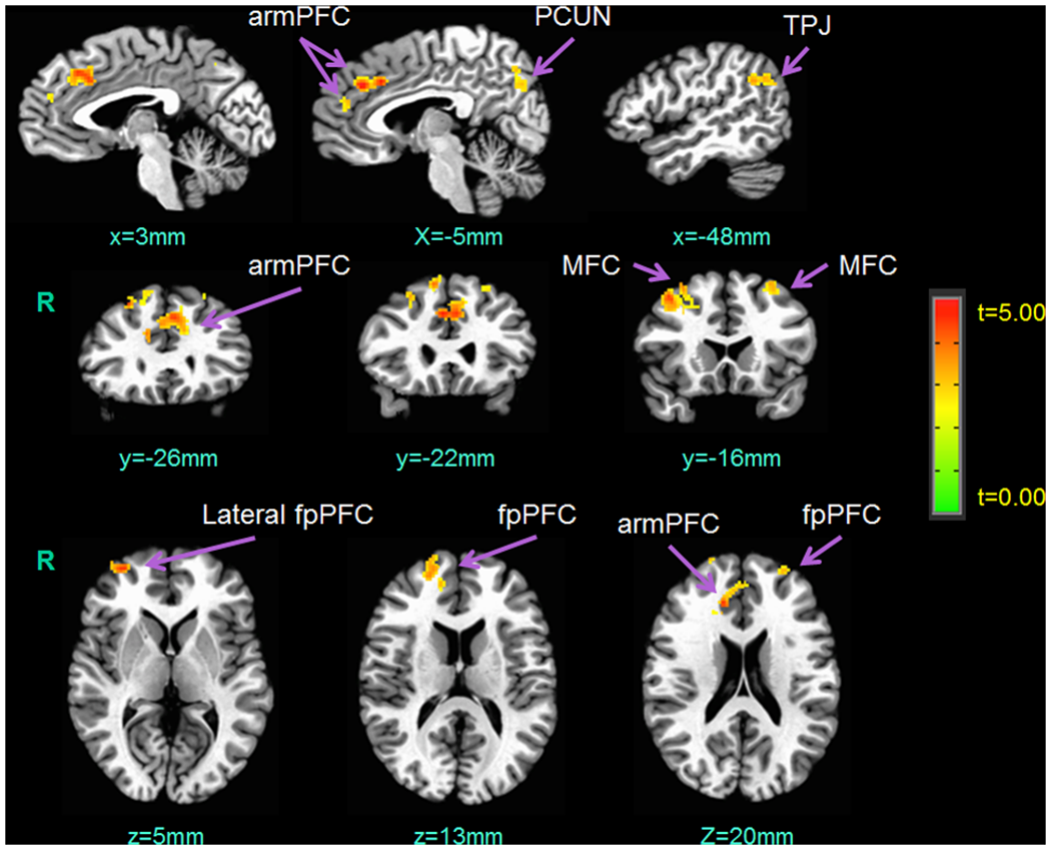
The main effects of the choices of true advice (relative to false advice) on functional brain activation when subjects played the role of the Sender. R = right hemisphere; armPFC = anterior rostral medial prefrontal cortex; PCUN = precuneus; TPJ = temporoparietal junction; MFC = middle frontal cortex; fpPFC = frontopolar prefrontal cortex.

**Fig 3:**
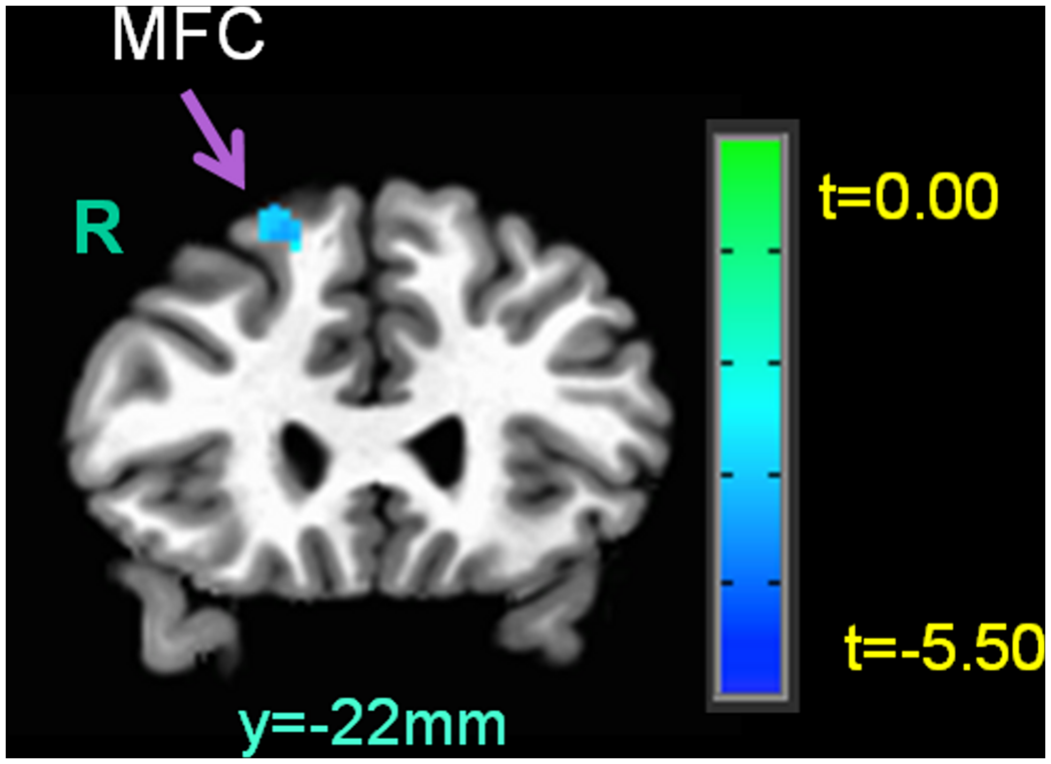
The main effects of the total RTI score on functional brain activation when subjects played the role of the Sender. R = right hemisphere; MFC = middle frontal cortex.

Our LME results of the whole-brain analysis revealed that there were main effects of advice choice, as well as the standardized RTI score, on functional brain activity (see Table 3). Specifically, when subjects served as the Sender and chose true advice, the following brain regions showed increased hemodynamic activity: bilateral anterior rostral medial prefrontal cortex (armPFC), bilateral middle frontal cortex, right temporoparietal junction (TPJ), bilateral frontopolar prefrontal cortex (fpPFC), and right precuneus (see Fig 2). Furthermore, when the subjects exhibited a high RTI score, they showed decreased right middle fronto-cortical activity during decision-making as the Sender (Fig 3). Finally, there was no interaction effect between the advice choice and the RTI score.

**Table 3:** Linear Mixed-Effects Models of the Whole-Brain Analysis of BOLD Responses in Relation to the Advice Choice and Risk Propensity (*N* = 13).

Brain region	BA	*β*	*F*	TC X	TC Y	TC Z	Cluster size
**Main effect of the advice selection (AS)—true advice vs. false advice**							
**Bi Anterior rostral medial prefrontal cortex**	9/32	0.15	27.60**	−6.6	−32.8	32.5	695
**R Middle frontal cortex**	6/8	0.24	21.18**	−38.4	−15.9	45.6	415
**R Temporoparietal junction**	39/40	0.11	14.59**	−47.8	51.6	28.8	106
**R Frontopolar prefrontal cortex**	10	0.19	16.56**	−17.8	−55.3	11.9	101
**L Middle frontal cortex**	6/8	0.10	12.81**	29.1	−15.9	49.4	80
**R Lateral frontopolar prefrontal cortex**	10	1.11	20.03**	−30.9	−53.4	6.2	77
**R Precuneus**	7	0.13	14.77**	−0.9	64.7	41.9	71
**L Frontopolar prefrontal cortex**	9/10	0.24	16.88**	29.1	−53.4	26.9	65
**Main effect of the total RTI score (RTI)**							
**R Middle frontal cortex**	6/8	−0.05	22.76**	−21.6	−21.6	51.2	73
**Interaction of AS × RTI**							
**No interaction effect**							

**Significant at *p* < .005 and 65 voxels. *df* = 1, 604. *β* = the peak standardized coefficient within clustered activation. Talairach coordinate (abbreviated as TC) represents the location showing the peak *F*-statistic of clustered activation. Bi = bilateral, L = left hemisphere, R = right hemisphere, BA = Brodmann area.

### Functional MRI analysis of the amygdala

**Fig 4:**
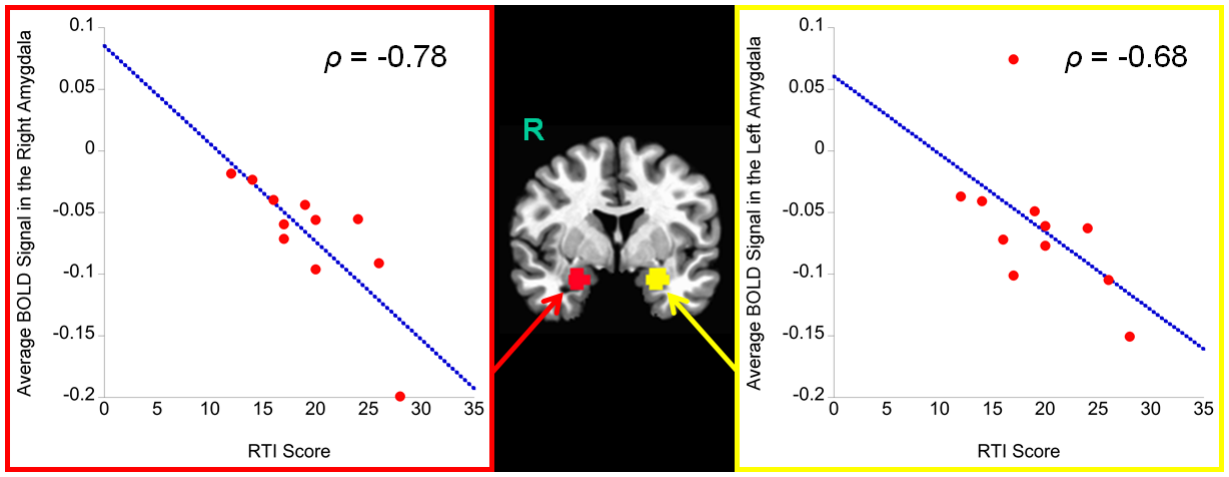
The effects of the RTI score on functional amygdala activation during decision-making of advice when subjects played the role of the Sender. The y-axis represents *β* coefficients. R = right hemisphere.

Table 4 shows the results of amygdala activity during decision-making as the Sender. Note that two subjects were excluded from this analysis because they never chose false advice across trials. Our results yielded that the advice choice was not associated with bilateral amygdala activity. In contrast, the effect of the RTI score on amygdala activity (across hemispheres) was significant. Follow-up Spearman correlation indicated that the RTI score was negatively associated with amygdala activity (*ρ*(8) = −0.76 *p* < 0.01); when subjects, acting as the Sender, showed higher RTI score, they were likely to exhibit decreased amygdala activity during decision making, regardless of the advice choice or hemisphere (see Fig 4).

**Table 4:** Repeated measures ANCOVA of the Amygdala BOLD Responses in Relation to the Advice Choice, Risk Propensity, and Hemisphere (*N* = 11).

Source	*df*	*F*	partial *η* ^*2*^
**Between subjects**			
**RTI score (RTI)**	1	11.98**	0.568
**Within-group error**	9	(0.00)	
**Within subject**			
**Advice selection (AS)**	1	2.33	0.200
**Hemisphere (H)**	1	0.14	0.000
**RTI × AS**	1	2.65	0.220
**RTI × H**	1	0.24	0.040
**AS × H**	1	0.00	0.000
**RTI × AS × H**	1	0.28	0.000
**Within-group error**	9	(0.00)	

*Note*: Values enclosed in parentheses represent mean square errors. RTI score was included as a covariate in the model.

**p < .01.

## Discussion

We conducted a novel exploration of functional brain responses to the choices of truth telling and risk propensity in asymmetric information. We hypothesized that when subjects performed as the Sender they would show functional brain changes, especially the amygdala, during the choices of true advice, depending on their risk propensity. The study revealed three major findings.

First, risk propensity was not significantly correlated with the choices of true advice. This was slightly surprising because previous studies have suggested the relationship between trusting behavior and risk propensity [1, 50]. Our lack of finding this correlation might be due to the small sample size. However, when each of different types of risk propensity were tested, recreational risk-taking type was significantly correlated with the choices of true advice. That is, the higher individuals showed in the recreational aspects of risk propensity (e.g., rock-climbing, scuba diving), the more likely they were to tell the truth to the partner. This is reasonable because some studies have reported that individuals with high recreational risks are inclined to show openness and curiosity for a novel situation [62, 63], which might motivate them to pursue adventurous interaction with a stranger and to tell the truth to the stranger. Future research needs to scrutinize the relationship between the specific domain of recreational risks and truth telling.

Whether subjects told the truth or a lie in asymmetric information was determined by not only the behavioral factor of recreational risk propensity but also the neurobiological factor of functional cortical activation, and this was our second major finding. When the subjects played the role of the Sender, some of their cortices showed increased functional activation in response to the choices of true advice. This cortical activation specifically included the bilateral armPFC, bilateral middle frontal cortex, right TPJ, bilateral fpPFC, and right precuneus. These findings suggest that the bilateral prefrontal cortex, as well as TPJ and precuneus in the right hemisphere, may be involved in the neural processing of truth telling under asymmetric information. Consistent with these fMRI results, previous studies using voluntary trust games have shown that increased activation in the armPFC is related to both reciprocity and trust [24, 25], and increased activation in the bilateral fpPFC and right TPJ is exclusively associated with trusting behavior [25]. Note that voluntary trust games assess trusting behavior under perfect information, where no players have more or less information to play the games than another. In contrast, our Communication Game measured truth-telling behavior under asymmetric information, where the Sender had superior information regarding available payment pairs and served as an advisor for the partner. In spite of these different relational contexts between voluntary trust games and the Communication Game, we obtained common neuroimaging evidence between the studies. In other words, our results extended previous findings, such as that the bilateral armPFC, bilateral fpPFC, and right TPJ are involved in not only decisions to trust in perfect information but also decisions to tell the truth in asymmetric information. This suggests the possibility that the bilateral armPFC, bilateral fpPFC, and right TPJ are general neuro-biomarkers for decisions to tell the truth in economic transactions.

The armPFC and TPJ are connected to each other anatomically [64, 65] and functionally [66], and these regions, along with the precuneus, have been considered as parts of the neural network for the mentalizing system [66–68] or theory of mind (ToM) [69–71], both of which refer to the ability to attribute, reason about, and represent the mental states of another person [72]. While the neural network of the mentalizing system/ToM consists of multiple brain regions [67, 69, 70], the armPFC, TPJ, and precuneus seem to be particularly important in philosophical reasoning and trust in communication, such as true/false belief reasoning [73], the beliefs in moral judgment [74], understanding and predicting other people’s intensions [75], and cooperation and deception [76, 77]. The current results of functional responses to the choices of true advice in the armPFC, TPJ, and precuneus seem to be consistent with the above findings, and they suggest that the neural mechanism underlying the mentalizing system/ToM presumably influences decisions to tell the truth in asymmetric information.

The above findings also suggest that dysfunctional armPFC, TPJ, and/or precuneus may be the sign of high risk for social communication problems regarding honesty and deception. For example, autistic children show atypically decreased responses to ToM tasks in the medial PFC and TPJ [78, 79], and exhibit impaired ToM-related behaviors [80, 81], such as “too much honesty/truth telling” [82]. Although speculative, these studies, as well as our current results, may suggest that increased PFC-TPJ responses to truth telling plays a role in executing context-appropriate moral judgments of truth telling.

In addition to the armPFC, TPJ, and precuneus, our results also identified greater functional activation in the fpPFC in both hemispheres during the choices of true advice. The fpPFC partly functions as the representation of a long-term goal-oriented sequence of multiple social events and rules [83], such as subgoal processing of problem-solving (i.e., procedural planning of achieving a first subgoal before higher-order subgoals are satisfied) [84]. Thus, our results might indicate that decisions to tell the truth were motivated at the base of the long-term prospects of consequences and benefits of repeatedly delivering true advice, which was reflected as greater activation in the fpPFC during the choices of true advice in the present study [25, 83].

Furthermore, we found increased activity in the bilateral middle frontal cortex when subjects chose true advice. A previous study has reported that this cortical area, especially Brodmann area 8, shows greater activation as individuals experience increased uncertainty in social events [85]. Our Communication Game indeed created some degrees of uncertainty in social interactions between the Sender and the Receiver, because, for example, the Sender was uncertain about whether the partner would follow her/his advice. Hence, our findings of increased activity in the middle frontal cortex in the Senders might reflect their uncertainty about the partner’s subsequent decisions to trust. However, our results additionally revealed that the right middle frontal cortex showed decreased activity in individuals with high risk propensity. This might reflect that high risk-takers were less concerned with uncertainty or a risk of a negative outcome, such as their advice being rejected by the partner, in the Communication Game than low risk-takers.

The third main finding was association of the amygdala response with risk propensity, regardless of the advice choice. When subjects were high in risk propensity, they bilaterally showed decreased amygdala activity during the decision-making of advice for the partner. Although this finding was not directly related to our primary interest in truth telling, it may suggest that low risk-takers tend to have increased amygdala activity during social interactions. The relationship between the amygdala and social perception has been reported, such that increased activation in the amygdala is found in response to untrustworthy faces [11–13, 15], although the relationship may not be monotonic [14, 15]. Therefore, it may be possible that, as compared to high risk-takers, low risk-takers show negative bias in social perception of the partner (e.g., as being more untrustworthy) during social interactions.

Alternatively, the difference in amygdala activity to the choices of advice between low and high risk-takers may reflect the intensity of their social vigilance. Previous studies have shown that increased amygdala activation is related to experience of increased social vigilance [12, 86]. In the present study, low risk-takers showed greater amygdala activation during the choices of advice than high risk-takers, suggesting that low risk-takers possibly faced more vigilance with the partner as compared to high risk-takers. In contrast, high risk-takers may show blunted amygdala response to such social vigilance. Future research needs to examine the relationship between risk propensity, social perception/vigilance, and functional amygdala activity during social interactions.

Therefore, our neuroimaging results revealed that truth telling was associated with increased activation in the armPFC, TPJ, precuneus, middle FC, and fpPFC. We suggest that these identified brain regions may be biomarkers for truth telling under asymmetric information, which can be applicable to practical settings. For instance, trust in a therapeutic relationship between a mental health practitioner and a patient, called therapeutic alliance, is critical to predict treatment success [8, 9, 29–33]. To assist building a therapeutic alliance, both practitioner and patient are required to tell the truth under asymmetric information. That is, while the practitioner needs to tell the truth/appropriateness about diagnosis, prognosis, and/or treatment, the patient also needs to tell the truth about their symptoms. By measuring and presenting brain activities (especially a practitioner’s activities) identified in the present study, it may be possible to overcome a patient’s skepticism and to convince the patient to follow the practitioner’s advice, which would contribute to the development of therapeutic alliance.

Although our results showed novel and significant implications in the neural activity for truth telling in the transaction of asymmetric information, the current study had a limitation. Our data were based on *N* = 13 subjects, which was not large sample. Thus, it may be necessary confirm our findings with a larger sample size, although the number of false positives should not be affected by a small sample size [87]. Another study limitation was that most subjects delivered true advice more often than false advice (see Table 1), which might slightly bias BOLD signal contrasts between the two conditions. This biased behavioral pattern is consistent with previous findings [26]. Similar to the above issue, we found that, when subjects acted as the Receiver, the majority of them overwhelmingly followed advice. Consequently, the present study made it difficult to obtain BOLD contrasts between following advice (86.1% of all trials on average) and not following advice (12.6% of all trials on average), although this analysis was additionally interesting. Future studies need to increase behavioral variance to balance the frequency of responses as the Sender and Receiver respectively, as well as focusing on functional brain activity during the performances as the Receiver.

In conclusion, the present study examined the brain activity in relation to truth telling and risk propensity under asymmetric information. It was found that increased frequency of truth telling was associated with increased recreational type of risk propensity. In addition, our LME models of the whole-brain analysis revealed that truth telling led to greater functional activation in the bilateral armPFC, bilateral fpPFC, bilateral middle frontal cortex, right TPJ, and right precuneus, while right middle fronto-cortical activity was additionally influenced by risk propensity. Finally, there was significant effect of risk propensity on amygdala response during decision-making of advice, such that low risk-takers showed elevated amygdala response. This study provided social implications regarding the neural system for truth telling in asymmetric information.

## Supporting Information

S1 Dataset Demographic and Behavioral Data. The variable for “sex” is coded as 1 = female and 2 = male. The variable for “education” is coded as 5 = Some college or technical school (at least one year), 6 = College graduate, and 7 = Graduate professional training (Masters or above).
(XLSX)

S2 Dataset Functional Whole Brain Data. The variable for “advice selection” is coded as -1 = false advice and 1 = true advice. All values of functional brain activity indicate average *β* of BOLD signals at the peak voxel within each identified region.
(XLSX)

S3 Dataset Functional Amygdala Data. The variable for “advice selection” is coded as -1 = false advice and 1 = true advice. All values of functional amygdala activity indicate average *β* of BOLD signals at the peak voxel within each hemisphere of the amygdala.
(XLSX)

S1 Table Probabilities of the Computer-Programmed Partner’s Responses. In the present study the player partner was actually performed by computer programming across all trials; the player partner was programmed to choose true/false advice or to follow/disregard advice with varying probabilities, depending on subjects’ response at the previous trial with details presented in S1 Table.
(DOCX)
