# Supplementary material for: Neural Responses to Truth Telling and Risk Propensity under Asymmetric Information
Source: PLoS One. 2015 Sep 1;10(9):e0137014. doi: 10.1371/journal.pone.0137014 (PMC4556667; doi:10.1371/journal.pone.0137014)
Supplement: S1 Table — In the present study the player partner was actually performed by computer programming across all trials; the player partner was programmed to choose true/false advice or to follow/disregard advice with varying probabilities, depending on subjects’ response at the previous trial with details presented in S1 Table. (DOCX) [file pone.0137014.s004.docx]

Supporting Information

**Neural Responses to Truth Telling and Risk Propensity under Asymmetric Information**

Hideo Suzuki^1^, Masaya Misaki^1^, Frank Krueger^2^, and Jerzy Bodurka^1,3^

In the present study the player partner was actually performed by computer programming across all trials; the player partner was programmed to choose true/false advice or to follow/disregard advice with varying probabilities, depending on subjects’ response at the previous trial with details presented in the Supplemental Table S1.

**Supplemental Table S1**. Probabilities of the Computer-Programmed Partner’s Responses.

|  | | Computer-programmed partner’s response at next trial | |
| --- | --- | --- | --- |
|  |  | Probability of choosing true advice : Probability of choosing false advice  (if Sender) | Probability of following advice : Probability of disregarding advice  (if Receiver) |
| At previous trial, if a subject was… | Sender,  choosing true advice | 0.65 : 0.35 | 0.63 : 0.37 |
|  | Sender,  choosing false advice | 0.38 : 0.62 | 0.39 : 0.61 |
|  | Receiver,  following advice | 0.52 : 0.48 | 0.55 : 0.45 |
|  | Receiver,  disregarding advice | 0.55 : 0.45 | 0.50 : 0.50 |

*Note*: Values indicate the probabilities that the computer-programmed partner chose true/false advice (if Sender) or following/disregarding advice (if Receiver). The probabilities varied, depending on subjects’ responses at the previous trial.
